# Supplementary material for: Sleeping with time in mind? A literature review and a proposal for a screening questionnaire on self-awakening
Source: PLoS One. 2023 Mar 23;18(3):e0283221. doi: 10.1371/journal.pone.0283221 (PMC10035927; doi:10.1371/journal.pone.0283221)
Supplement: S1 File — (PDF) [file pone.0283221.s001.pdf]

Supporting Information

1. SAQ and MEQ

S1 Table. Responses to each SAQ question (number of respondents and percentage of the group total) divided by groups according to the MEQ scores.

SAQ question		Assigned score	All (n = 153)	MEQ-based group		
				Morning (n = 16)	Intermediate (n = 100)	Evening (n = 37)
**Q1**	yes	1	150 (98%)	16 (100%)	97 (97%)	37 (100%)
	no	0	3 (2%)	0 (0%)	3 (3 %)	0 (0%)
**Q2**	Every day	0	3 (1%)	0 (0%)	2 (2 %)	1 (3 %)
	At least once a week	0	61 (41%)	10 (63%)	36 (36%)	15 (41%)
	Only on nights before important events (Which ones? Please specify)	3	89 (58%)	6 (38%)	62 (62%)	21 (57%)
**Q3**	Yes	0	65 (42 %)	5 (31%)	47 (47%)	13 (35%)
	No, just in the morning before the alarm clock	3	88 (58 %)	11 (69%)	53 (53%)	24 (65%)
**Q4**	5 minutes before or less	3	32 (21%)	1 (6%)	25 (25 %)	6 (16%)
	Between 5 and 15 minutes	2	60 (38%)	12 (75%)	32 (32%)	16 (43%)
	Between 15 and 30 minutes	1	48 (31%)	3 (19 %)	32 (32 %)	13(35%)
	More than 30 minutes (please specify …………………..)	0	13 (8%)	0 (0%)	11 (11 %)	2 (5%)
**Q5**	When you have to wake up at the usual time (e.g., 7 a.m. on a working/school day)	0	117 (76 %)	14 (88 %)	72 (72%)	31 (84%)
	When you have to wake up at an unusual time (e.g., 3 a.m.)	3	36 (24 %)	2 (12%)	28 (28%)	6 (16%)

2. SAQ Italian version

SELF-AWAKENING QUESTIONNAIRE
VERSIONE ITALIANA

In particolari circostanze, per esempio prima di un esame universitario o un importante incontro di lavoro, alcune persone sono in grado di svegliarsi spontaneamente prima del suono della sveglia o addirittura non hanno bisogno di una sveglia. Le seguenti domande mirano a caratterizzare questo comportamento.

Istruzioni

Leggi ogni domanda con attenzione.
Scegli la risposta che caratterizza con più precisione la tua esperienza personale.
Per favore rispondi a **TUTTE** le domande.

1. Ti è mai capitato di svegliarti volontariamente ad un orario prestabilito senza l'ausilio di una sveglia o altri aiuti esterni?
   - ❑ Si
   - ❑ No

2. Se si, quanto frequentemente ti succede?
   - ❑ Tutti i giorni
   - ❑ Almeno una volta a settimana
   - ❑ Solo prima di un evento importante (Quale evento? Specificare: …………………………………………………………………………………………… …………………………………………………………………………………………… ……………………………………………………………………………..)

3. In tali occasioni hai dei risvegli frequenti durante la notte o ti svegli solo poco prima dell'orario prestabilito?
   - ❑ Mi sveglio più di una volta (specificare in media quante volte: ………….)
   - ❑ Mi sveglio solo una volta poco prima dell'orario prestabilito

4. Quanti minuti, in media, ti svegli **prima** dell'orario prestabilito?
   - ❑ 5 minuti prima o meno
   - ❑ Tra 5 e 15 minuti
   - ❑ Tra 15 e 30 minuti
   - ❑ Più di 30 minuti (specificare quanto ……………………………………...)

5. Nella tua esperienza, è **piu' probabile** che tale risveglio spontaneo si verifichi:
   - ❑ Quando ti devi risvegliare ad un orario abituale (per esempio, 7 del mattino in giornata lavorativa/scolastica)
   - ❑ Quando ti devi risvegliare ad un orario insolito/non abituale (per esempio, 3 del mattino)

3. Description of battery tests and their scores

1. ISI is a 7-item self-report questionnaire to evaluate subjective symptoms and daytime consequences of insomnia as well as the degree of distress caused by these difficulties. Higher scores indicate greater insomnia severity. The total score ranges are: no significant insomnia (0–7), sub-threshold insomnia (8–14), moderate insomnia (15–21), and severe insomnia (22–28). In the literature. “responders” to treatment are defined by a decrease of ≥8 points on the ISI total score, whereas “remitters”xw are determined by a total ISI score <8 at the end of the treatment. α = 0.75.

2. The MEQ-SA is a 19-item self-report scale designed to evaluate subjective circadian typology: morning-, intermediate- and evening circadian preference subjects. The total score ranges from 16 to 86. α = 0.68.

3. The PSQI is a 19-item self-report questionnaire used for a retrospective evaluation of sleep quality over the previous months. Seven clinical domains regarding sleep problems are assessed: sleep quality, sleep latency (SL), sleep duration, habitual SE, sleep disturbances, use of sleeping medications, and daytime dysfunction. All the items of the questionnaire are scored, and a single factor of global sleep quality is then computed. A total score 5 is considered an indicator of bad sleep quality

4. The PSWQ is a 16-item instrument aimed to measure the trait of worry. Each item is rated on a scale of one (“not at all typical of me”) to five (“very typical of me”), with items 1, 3, 8, 10, and 11 that are reverse scored. A total score of 50 has been identified as a reliable cut-off to discriminate generalized anxiety disorder patients from healthy subjects with both a high specificity (90%) and sensitivity (82%).

5. The STAI-Y is a 40-item self-report scale based on a 4-point Likert scale designed to evaluate anxiety (STAI-Y-1 = state anxiety, and STAIY-2 = trait anxiety in two different modules). STAI-Y scores > 44 in state module and > 41 in trait module are evaluated as clinically significant. α = from 0.86 to 0.95.

6. The Center for Epidemiological Studies-Depression (CES-D) is a 20-item measure that asks to rate how often over the past week one experienced symptoms associated with depression, such as restless sleep, poor appetite, and feeling lonely. Response options range from 0 to 3 for each item (0 = Rarely or None of the Time, 1 = Some or Little of the Time, 2 = Moderately or Much of the time, 3 = Most or Almost All the Time). Scores range from 0 to 60, with high scores indicating greater depressive symptoms

7. The RRS includes 22 items characterizing responses to depressed moods that are divided in three types: self-focused responses, symptoms focused responses, and responses focused on the possible consequences and causes of the mood. Every item must be rated on a scale from one (almost never) to four (almost always).

8. The PSAS is composed by 16 items, each rated on a 5-point scale that describes symptoms of arousal at bedtime. Eight items evaluate cognitive arousal and eight evaluates somatic arousal. Higher scores suggest higher pre-sleep arousal.
