# Supplementary material for: Sleeping with time in mind? A literature review and a proposal for a screening questionnaire on self-awakening
Source: PLoS One. 2023 Mar 23;18(3):e0283221. doi: 10.1371/journal.pone.0283221 (PMC10035927; doi:10.1371/journal.pone.0283221)
Supplement: Attachment — Submitted filename: PLOS response to reviewers.docx [file pone.0283221.s002.docx]

Dear Dr. Verga,

Thank you for submitting your manuscript to PLOS ONE. After careful consideration, we feel that it has merit but does not fully meet PLOS ONE’s publication criteria as it currently stands. Therefore, we invite you to submit a revised version of the manuscript that addresses the points raised during the review process.

Please address all comments raised by the reviewer.

We thank the editorial office and the anonymous reviewers for their consideration of our manuscript, and for the constructive feedback. We have been working extensively to respond to the reviewers’ concerns, which we addressed in full, leading to a significant improvement in our manuscript. We additionally reviewed the manuscript and introduced minor changes to improve readability. Please find our point-by-point responses below. We sincerely hope that the current version of our paper will be further considered for publication in PLOS ONE.

Reviewer #1: Summary:

The paper by Verga et al. titled “Sleeping with time in mind? A literature review and a proposal for a screening questionnaire on self-awakening” investigates the ability to self-awake at a pre-set time without external help, i.e. the ability to end one’s sleep episode voluntarily. The authors provide a systematic literature review following the PRISMA guidelines, summarise the results of the selected studies, present a working definition for “self-awakening” and present their own screening questionnaire they developed based on the literature review. Their questionnaire was designed in English and translated to Italian and administered to 164 students at Maastricht University.

Altogether, the authors find substantial diversity in the 17 reviewed studies in terms of quality, study design and methods. Furthermore, the concept of “self-awakening” has been operationalised and framed differently across studies and the authors present a working definition for this phenomenon. Based on the review and their own study results, the ability to self-awake does not seem to be a common phenomenon albeit this results is very much limited by the small sample sizes across reviewed studies.

The study has several strength which are the adherence to PRISMA guidelines, access to the data, clarification and proposal of the concept behind “self-awakening”, clear language and writing, nice presentation of the results and a succinct summary. Limitations/weaknesses include the missing PRISMA flowchart and no detail on the quality of the reviewed studies despite this being mentioned, low sample size in their own study, raw data only available upon request and in general a relatively superficial handling and discussion of the phenomenon, possible mechanisms, underlying neurobiological facts or discussion of other known facts from related fields, such as time keeping mechanisms when awake.

I recommend major revisions which have to include a more in-depth discussion of the phenomenon including possible mechanisms.

We thank the reviewer for the overall positive evaluation of our work and the constructive feedback. We worked extensively on the manuscript to address the reviewer’s concerns and comments. In addition, we inserted the PRISMA flowchart as suggested in Figure 2, also reported here. The raw data (including the database for the systematic review, quality judgements, and detailed research results) and script have been uploaded in a publicly available repository. Furthermore, we have been reworking the discussion, which is now more in-depth.

For the detailed comments, please see the detailed point-by-point responses below.

**Fig. 2.** PRISMA flow diagram summarising the selection procedure.

Major issues:

Introduction:

1. The authors seem to make the point that sleep is still viewed as an inactive brain state. This view is outdated and I advise to discuss this more in light of the current evidence concerning sleep architecture, possibly functions of sleep, circadian mechanism/sleep homeostatic components, and cognitive aspects of sleep and during sleep

We thank the reviewer for their comment. Accordingly, we removed the statement concerning the view of sleep as an inactive state and added a more updated statement.

Lines 62-67: “*Human sleep is a complex physiological function. Behaviourally, it alternates with waking state and is characterised by an increased threshold to sensory input, reduction of motor output, typical changes in central and peripheral physiology, and diminished conscious awareness (Stickgold and Walker, 2009). Importantly, from a psychological standpoint, sleep plays a crucial role in the regulation of daytime cognitive functions like memory and emotion (Walker et al., 2009; Tempesta et al., 2018; Klinzing et al., 2019).*”

2. Please add references to the claim that habitual self-awakening, i.e. waking up at the same time every day, is indeed driven by circadian mechanism. I’m unsure whether the evidence is clear on this.

We thank the reviewer for raising this very important point. Although there is no clear evidence on the topic, this represents a crucial argument in our framework. As depicted in figure 1 the distinction between habitual self-awakener and those who use self-awakening as the ability to possibly perform an awakening at any time during the night is fundamental. Indeed, the former case is likely more frequent (15.1% reported by Malloggi et al., 2021 compared to the 6% in our own sample) and, as also pointed out by reviewer 2, it may be a learned behaviour involving circadian rhythm issue and not be ascribable to the complex cognitive activity described in figure 4.

Malloggi S, Conte F, Albinni B, Gronchi G, Ficca G, Giganti F. Sleep and psychological characteristics in habitual self-awakeners and forced awakeners. Chronobiol Int. 2022 Apr;39(4):547-556.

3. In general, more references and in-depth background to the current topic is warranted. For example, what could be the biological differences between habitual self-awakening and self-awakening ability and so on?

The issue regarding the biological differences between habitual self-awakening and self-awakening ability has not been investigated in depth at all. The specific distinction between these two subtypes comes from theory and knowledge about sleep and circadian system and common sense. Indeed, it is unlikely that an habitual self-awakener may wake up at an unusual time, and that this could be reconducted to complexity of the phenomenon explained in figure 4 (likely involving long term memory, working memory, central executive, ultradian timekeeper, and regained volition during a minimal state of consciousness). Instead, habitual self-awakeners may likely synchronise themselves with circadian cues, such as body temperature and dissipation of sleep pressure, which are able to increase the probability of an awakening (in particular during usual wake-up time) throughout mechanisms related to the interaction between circadian timer and learned behaviour. In absence of relevant studies, these remain largely speculations. We hope that future studies may target these issues more directly.

Methods:

4. Could the authors please provide information about the program used for statistical analyses, including the version number and used packages if applicable

We apologise for the lack of this crucial information in the previous version of the manuscript. This has now been specified at lines 230-231: “*All statistical analyses were conducted in R (version 4.1.2, https://www.R-project.org/; R core team, 2020) running in R studio (version 1.3.959, http://www.rstudio.com/, R studio team, 2020).*”

The corresponding references were added to the reference list.

5. The data are not really openly available, they are only available upon request. Why?

Thank you for noticing this issue. The data (both for systematic review and SAQ) and scripts for the current manuscript have been uploaded in OSF at the following link (https://osf.io/kaunw/) and are publicly available.

6. Could the authors please clarify what is meant by “NEAR/0” (lines 120-128)

The proximity operator NEAR/0 is used in database research to find records where the terms joined by the operator are within a specified number of words of each other (in this case, adjoint).

Lines 129-131: “*Search operators (e.g., AND; the proximity operator NEAR/0 was employed to find records where the search terms joined by the operator were adjacent) were used to combine the following key-terms: (...)”*.

7. It is unclear why each study could obtain a max. of 13 points (line 152). Please clarify.

We thank the reviewer for raising this point that needed to be clarified. Each study could obtain a maximum of 14 points; however, the maximum quality score obtained by the selected studies was 13. In accordance with the reviewer's suggestion, we revised the sentence as follows: “*Each study could obtain a maximum of 14 points; the average quality score for the selected studies was 10.71 (median = 11; range = 9-13).*”

Additionally, please note that the CASP questionnaire is intended as an additional quality tool, primarily intended for educational purposes; thus, a fixed scoring system is not provided by the authors (for details see <https://casp-uk.net/casp-tools-checklists/> )

8. Cohen’s kappa for the inter-rater reliability was rather low, please comment on the difficulties.

The studies retrieved through our systematic review were characterised by a very high heterogeneity. As we discuss in the manuscript, this affected both the theoretical and methodological approach adopted to study the phenomenon of self-awakening. The low inter-rater reliability likely reflects these inconsistencies, especially at the methodological level.

9. It in unclear how the sampling procedure for their own study was done, nor why they stopped at a certain amount of participants

Participants self-recruited for the study by either contacting the experimenters directly (we advertised the study on the bulletin boards in campus) or through the SONA system of Maastricht University. SONA is a participant pool management system used by many universities (https://www.sona-systems.com/), where available studies can be easily found by students. Usually, students can select a study of their choice to partake in, in exchange for university credits or cash.

The recruiting procedure stopped for both practical as well as theoretical reasons. First, given the paucity of previous work on self-awakening and the exploratory nature of our study, we could not perform an a-priori power analysis. Yet, a number of participants > 150 can be considered a reasonable sample size for most types of analyses, especially for simple statistics as the ones performed in the current study. Recruitment stopped as no further participants would sign up after a waiting period of 2 months since the last recruitment, at which point we had to remove the study from the SONA system as our allowed period was terminated.

We specified the details of participants’ recruitment at lines 183-190: “*The questionnaire was administered to 164 students at Maastricht University (Maastricht, the Netherlands); all students were enrolled in the English track and used English daily as their main study language. The study was advertised locally on bulletin boards across campus and on the SONA system of Maastricht University. Interested participants could either contact the experimenters directly via email or could select the study from those available on the SONA system. All students who completed the battery were compensated with university credits. Ethical approval was granted by the local ethical committee of Maastricht University, Faculty of Psychology and Neuroscience (agreement nr. OZL_207_04_04_2019).*”

Please note that we slightly rearranged this session to improve readability and cohesion.

10. Why was the cut-off for their SAQ at 11; Could the authors provide an explanation?

The cut-off was set based on our theoretical definition of self-awakener and the distribution of the scores; in general, a higher score indicates a higher probability of being identified as a self-awakener by the questionnaire. More specifically, within each question, answers related to higher score correspond to features likely associated with a self-awakener (See Table 1, Supporting Information). The combination of the scores to be correctly identified as a self-awakener is at follows:

- Question 1: necessary answer = “yes”, 1 POINT
- Question 2: “only on nights before important events”, 3 POINTS
- Questions 3: “No, just in the morning before the alarm clock”, 3 POINTS
- Question 4: the answer to this questions is stratified; a potential self-awakener could obtain a score of 1, 2, or 3 POINTS
- Question 5: “When you have to wake up at an unusual time”, 3 POINTS

By summing all the points, we can see that the minimum score to be defined as a self-awakener is 11 (Q1+Q2+Q3+Q4+Q5 = 1 + 3 + 3 + 1 + 3).

We added an explanation at lines 207-208: “*We set the cut-off to define a self-awakener at 11/13; a score of 11 is the minimum score corresponding to a potential self-awakener, based on the score distribution in the SAQ items.*”

11. Lines 191-195: More information is needed to understand what was actually carried out: 1 analysis, 9 separate analyses, or something completely different?

Thank you for allowing us to clarify this important detail of the analyses. We rephrased as follows (lines 194-197): “*To evaluate possible differences between these two groups (self-awakeners vs. non-self-awakeners), separate non-parametric Mann-Whitney tests were performed using the outcome of each test of the battery as dependent variable*.”

12. SAQ “Aim” section: The information given under “aim” in Table 1 points towards “night before a test or important meeting” -> this is confounded by possibly anxiety feelings or feeling nervous; yet the authors suggest that they are probing for self-awakening explicitly without underlying conditions such as stress or anxiety. Could the authors clarify why they think this is still valid?

We believe that non-pathological levels of anxiety and worry may be part of the phenomenon; however, these should not result in an increased amount of nocturnal awakenings that clearly affect the validity of the construct (i.e. awakenings during which one could check time and moment of the night).

We investigated this issue by means of question 3 of the SAQ “When you experience these spontaneous awakenings, do you wake up more than once throughout the night?”; therefore, a self awakening should not be directly reconducted to disrupted sleep caused by stress, anxiety, or other causes.

13. SAQ Q2 and Q5: I think that a neutral answer option should be given here, e.g. “neither” (Q5) and the scale is not adequate in Q2 (every day vs at least 1/week or only on nights before important events). What about other timescales?

Thank you for the valuable suggestions. Regarding Q2, we selected the most representative time-scales based on the existing literature. Longer time-scales (e.g., once a year or longer) would characterise sporadic and unpredictable phenomena, not consistent with an ability to self-awaken spontaneously.

Regarding Q5, the two answers provided are mutually exclusive. Because of this, the response “neither” would imply that these participants are not able to wake up spontaneously, thus contradicting a possible response “yes” to Q1.

Results:

14. Please clarify what is meant by “ps” (line 298) and “rs” (line 301). Multiple p values and r values? If so, maybe giving a range would be more appropriate.

Indeed, “ps” was intended as a plural form of p-value. To avoid confusion, we rephrased as “ (all p-values > .44)”

“rs” indicate Spearman rho, as per APA guidelines. To avoid confusion, we now report the “s” as subscript, in the form: r_s_

15. Lines 305: It is unclear to me what the MANOVA actually tested; what were the dependent and what the independent variables?

Please note that in response to a comment from Reviewer 2, the MANOVA was discarded.

16. Lines 306-314: Could the term “univariate test” be replaced with the actual name of the test that was conducted?

Because we removed the MANOVA, the corresponding univariate tests were also discarded.

17. Fig 2:

We updated the figure based on the reviewer’s feedback. The new figure is reproduced here with the corresponding caption:

**Fig. 2*.*** Differences between Self-Awakeners and non-Self-Awakeners by test scores. The difference between groups was non-significant for all measures. For all boxplots, each box contains the interquartile range (i.e., the range containing 50% of all observations) and the median value (middle line), while the whiskers represent values extending outside 50% of the observations. Abbreviations: ISI = Insomnia Severity Index; MEQ = Morningness-Eveningness Questionnaire; PSQI = Pittsburgh Sleep Quality Index; PSWQ = Penn State Worry Questionnaire STAI = State-Trait Anxiety Inventory; BDI = Beck Depression Inventory RRS = Ruminative Response Scale; PSAS= Pre-Sleep Arousal Scale; CES = Center for Epidemiologic Studies Depression Scale (see Supporting Information for details).

The colour-coding legend “self-awakener” is currently 0 and 1 without explanation. I suggest to change to yes/no instead.

Concerning the lack of explanation, the dummy variable 0/1 is typically employed to indicate the presence or absence of the quantity indicated in the legend (in this case, whether the participant in a given group was a self-awakener or not). Yet, we agree that yes/no may be a more intuitive labelling, and we changed the legend labels accordingly (see Figure 2 above).

Also, I strongly recommend to plot the underlying data points, report the sample size per item if not the same across all plots and give an explanation of the errors bars (SD?).

- We modified the figure to include individual data points.
- The sample size is constant, as all subjects had to complete all tests.
- Regarding the error bars, we report median and IQR range, as it is standard practice for boxplots. This information is now also specified in the Figure caption, as follows: “*For all boxplots, each box contains the interquartile range (i.e., the range containing 50% of all observations) and the median value (middle line), while the whiskers represent values extending outside 50% of the observations.*”

The individual tests are only reported with abbreviations and should be explained in the Figure legend.

We now explained the abbreviations in full in the figure caption; additional information on each test is also reported in the Supporting Information.

18. Fig. 3: Similar to Fig. 2, underlying data points to be added, as well as a description of the error bars and sample size.

Please find reproduced here the updated figure and its caption. Please note that in response to a following comment, we also changed the number of ticks on the y-axis.

**Fig. 3.** Distribution of the SAQ scores depending on the respondent’s circadian preference group. Each boxplot contains the interquartile range (i.e., the range containing 50% of all observations) and the median value (middle line), while the whiskers represent values extending outside 50% of the observations.

19. Could it be that in S1 Q5 the 2 numbers (36 vs 117) in the “all” column gut confused?

Thank you for the careful reading and for spotting this mistake; this has now been corrected, and the entire table has been re-checked.

Discussion:

19. Line 355 talks about the seminal work of Child but does not explain in detail what it was (this was first presented in the Introduction but could be repeated here for clarity)

In accordance with the suggestion of the reviewer we add a brief sentence to report Child’s finding at line 393-394: “Although self-awakening is frequently observed in the general population, as reported in the seminal work by Child (1892), where 59% of the participants (200 in total) claimed this ability, (...)”.

20. In general, the discussion is nice to read and succinct. I feel though that it is substantially missing a lot of background information, especially on the context of circadian influences, possible mechanisms, time keeping mechanisms that happen outside of sleep, theoretical underpinnings etc. I strongly urge the authors to provide more information on possible mechanisms

We thank the reviewer for this comment. We added a paragraph in the discussion in which we further elaborate on these aspects.

Lines 421-435:”Neural mechanisms underlying self-awakening are far from being understood. Studies investigating polysomnographic alterations right before self-awakening reported discrepant results. Although it could be possible that the attempt to awake at predetermined time during the night could lighten sleep by reducing slow waves, previous findings do not clearly support this view. Born and collaborators (1999) found that time spent in different stages, sleep stages’ time distribution, and arousals showed no alteration. Similarly, also Zung and Wilson (1971) reported no significant difference in sleep macrostructure between control and experimental nights. Remarkably, a study by Aritake and colleagues (2012) investigated the neural correlates of self-awakening through near infrared spectroscopy (NIRS) and polysomnography. They also found no differences in any sleep stages between experimental and control conditions. However, they found an increase in oxyhemoglobin in the right prefrontal cortex and a decrease in delta power 30 minutes before the self-awakening. The increase of oxyhemoglobin in this area is important since some studies reported its involvement in time recognition (Maquet et al., 1996; Coull et al., 2004). This finding suggests the importance of the activation of brain areas related to time estimation abilities and the disengagement from deep sleep in order to successfully perform self-awakening.”

21. Lines 416-421: this section needs more additional references and in depth-description of the underlying mechanisms

Please see the previous comment and our response, where we discuss more about the possible mechanisms.

22. The discussion does not contain a limitation section. Could the authors reflect critically on their work and mention some limitations?

We agree with the reviewer on the importance of adding a limitation section, which has now been introduced at page 15, lines 497-510.

“*Lastly, we put forward some limitations of the current research. First, as stated in the introduction, there are relatively few studies on self-awakening; consequently, the conclusions that can be drawn from our systematic review are preliminary. We sincerely hope that the current manuscript may stimulate further research and lead to a more in-depth understanding of this fascinating phenomenon. Second, the number of self-awakeners compared to non-self-awakeners in our student sample was rather low: Because of the lack of a screening instrument and the relatively low percentage of self-awakeners in the general population, we could not select directly self-awakeners to match non-self-awakeners, nor was it possible for us to test indefinitely. While we partially addressed this issue with our statistical approach, the development of a screening questionnaire–such as the one presented here–may tackle this problem more effectively, by allowing researchers to pre-select and match groups of non- and self-awakeners. Third, our study lacked an objective sleep evaluation to confirm whether the self-awakeners identified by SAQ were indeed capable of spontaneously waking up at a desired time. Objective sleep evaluation by means of PSG is of paramount importance in the study of self-awakening, and we strongly advise future studies to include these measurements.*”

23. The working definition of their concept should be discussed more carefully in this discussion. What does it contain? What does it not contain? What could the underlying mechanisms be? Why is it so difficult to find a common definition, and so on…

We added a new paragraph in the discussion section discussing available findings regarding putative mechanisms (see above). However, the current manuscript describes a review based mostly on behavioural papers, and we strongly believe that an even more in depth discussion of biological mechanisms would be highly speculative.

\#####

Minor issues:

Introduction:

23. Lines 92-96: The authors could expand this section to meet my recommendations above: this is an interesting observation and provides first hints towards possible mechanisms

In accordance with the suggestion of the reviewer we added a brief sentence suggesting a possible physiological mechanism involving the disengagement from slow wave sleep. This issue is then further discussed in the discussion section.

Lines 100-102: “Accordingly, one possible explanation for the phenomenon of self-awakening might involve subjects’ ability to disengage from slow wave sleep (Aritake et al., 2012).”

24. Line 88: 51% of participants - what was the overall sample size?

The total sample size was 200. Please note that upon re-reading the manuscript, we need to rectify the percentage originally reported, which is 59 instead of 51%.

We amended the sentence at lines 92-93: *“The author found that 59% of the participants (200 in total) reported waking up at a predetermined time without disturbed sleep (Child, 1892).*”

25. Lines 101-102: It would be nice to discuss the concept of volition. What is it? What are the brain mechanisms involved? How would this be related to certain sleep stages etc?

We thank the reviewer for this suggestion. We surely agree that it would be nice to discuss such a complex concept. When considering sleep one can speculate that a regained volition could be present in a specific and peculiar state such as that of lucid dream. For example, it has been reported that specific brain areas (dorsolateral prefrontal and frontopolar cortices, parietal lobules, and inferior/middle temporal gyri) showed a significant increase in lucid dreaming compared to non-lucid dreaming during REM sleep (see Dresler, M., et al 2014. Volitional components of consciousness vary across wakefulness, dreaming and lucid dreaming. *Frontiers in psychology*, *4*, 987). Although interesting, we chose not to insert this part in the introduction since we consider this too speculative for the aim of our work and reviewer 2 was doubtful regarding the brief part regarding lucid dreaming in the introduction.

Methods:

25. Lines 120-121: Unfortunately, the search is a little bit outdated, maybe the authors should consider re-doing this to check if new studies have been published meanwhile?

We repeated the research on October 6th, 2022. One additional paper was retrieved (S. Malloggi, F. Conte, B. Albinni, G. Gronchi, G. Ficca & F. Giganti (2022) Sleep and psychological characteristics in habitual self-awakeners and forced awakeners, Chronobiology International, 39:4, 547-556, DOI: 10.1080/07420528.2021.2003375). The search date has been updated in the manuscript. However, please note that this paper targeted ***habitual*** self-awakeners; as stated by the authors “participants were classified as habitual SAs if they answered “I never use external devices” to the first question and/or “I always wake up before the alarm goes off” to the second. Based on our taxonomy (see Fig. 1 in the text), this is a different phenomenon compared to self-awakening ability, which is the focus of our manuscript. For this reason, the paper was not included in the systematic review. It was, however, cited in the manuscript when we explicitly mention habitual self-awakening (e.g., line 186).

26. Lines 120-130: Could the authors please present the search terms differently to facilitate reading?

We agree with the reviewer that the proposed presentation of search terms is rather cumbersome. However, we strongly believe that reporting the queries in their current form may facilitate the replication of our results, and therefore decided to leave the description as it was. However, to facilitate reading, we now specify what the search term stand for, at lines 129-131: “Search operators (e.g., AND; the proximity operator NEAR/0 was employed to find records where the search terms joined by the operator were adjacent) were used to combine the following key-terms: (...)”.

27. Lines 131: I don’t quite understand the argument concerning language bias: if only English publications were selected anyways, why does this reduced language bias?

We agree with the reviewer that this issue was not clearly laid out. We did not explicitly restrict the database searches to publications written in English. Often, peer-reviewed manuscripts in other languages would still include an English version of the abstract or title; this way, our search would have captured these publications without the need to search for key terms in all possible languages. This said, we only retrieved publications written in English. This means that either this is the only language in which publications on self-awakenings were produced OR that publications in other languages did not fulfil our database search criteria. In either case, the reduction of publication bias is meant to signify that we did not exclusively and a-priori excluded other languages. However, we agree that this point may be debatable, and we therefore rephrased the sentence concerning publication bias as follows (lines 140-143): “*We allowed the search to include manuscripts written in languages other than English; however, only English studies were retained in the final set of suitable papers based on the criteria outlined in the following section.*”

28. Line 139: What is meant with “non-experimental”?

Experimental studies are classically defined as studies in which the experimenter introduces a perturbation to evaluate its effects on the normal behaviour of the participants. As such, experimental studies are usually contrasted with observational studies, in which the experimenter merely observes a given behaviour.

29. Line 153: What was the median and the range?

The median was 11, with a range between 9 and 12.5. This information has been added at line 164.

30. SAQ: Please provide more information about the translation. How many people were involved? Was it back translated into English? Was a professional translator involved?

The test was originally created in Italian by the first and last authors (LV and AG), and translated into English. The translated version was then checked by two native speakers of American English and by several colleagues who daily use English as their main working language. The final version was then re-translated in Italian by the other co-authors.

31. Lines 171-176: please provide information about the individual scales and their interpretation. Do higher number mean more severe insomnia etc?

The requested information has been added to the Supporting Information, at paragraph 3, titled “**Description of battery tests and their scores”.**

32. Lines 184-189: The big difference in sample sizes between self-awakeners (n=9) and non self-awakeners (n=144) is problematic. Did the authors consider any measures to increase the sample size in general? On the very positive side, they used bootstrapping methods to account for this limitation.

We agree with the reviewer that this is certainly problematic. We did consider increasing sample size; however, because it is not possible to know a-priori whether a participant is a self-awakener, this would have required a substantial amount of time and an economical cost for which we could not account. To compensate for this shortcoming, we opted for a bootstrapping approach, which is typically employed in clinical settings, where sample size is a known problem (for example, see Verga et al., Frontiers in Psychology, 2021; Schwartze & Kotz, LCN, 2021). We now discuss this problem in the limitations section, as follows (lines 500-506): “*Second, the number of self-awakeners compared to non-self-awakeners in our student sample was rather low: Because of the lack of a screening instrument and the relatively low percentage of self-awakeners in the general population, we could not select directly self-awakeners to match non-self-awakeners, nor was it possible for us to test indefinitely. While we partially addressed this issue with our statistical approach, the development of a screening questionnaire–such as the one presented here–may tackle this problem more effectively, by allowing researchers to pre-select and match groups of non- and self-awakeners.*”

Results:

33. Did the authors of the reviewed studies try to explain underlying mechanisms? If so, please review them when reviewing the results of the reviewed studies.

To the best of our knowledge the only study aimed to directly investigate underlying mechanisms of self-awakening is the one by Aritake and colleagues (Aritake et al (2012). Increased cerebral blood flow in the right frontal lobe area during sleep precedes self-awakening in humans. *BMC neuroscience*, *13*(1), 1-10.). We added in the discussion a paragraph containing findings from this study (lines 421-435, described in response to a previous comment).

34. Line 254: Does the term “significantly higher” refer to a statistical test or does it just mean “much higher”?

Thank you for the careful reading and for noticing this unclear point. The term was intended as “much higher”, and it has been modified accordingly. Line 283-284 now reads: “These percentages are much higher than the 17% based on our calculations.”

35. Lines 256-261: I don’t understand how the 8 papers which looked at gender differences found no gender difference but “within each paper’s self-awakeners’ samples, percentages of females and males […] showed a predominance of studies with a higher % of females compared to males”. What is the difference? How do these 2 sentences come together?

Thank you for pointing out this unclear description. The 8 papers did not specifically look at gender differences, but merely reported the number of males and females in their sample. The paragraph has been rephrased as follows (lines 285-291): “*Eight papers reported the number of males (n = 37) and females (n = 35) among their self-awakeners. These numbers indicate a no difference in the gender of self-awakeners when pooling across studies (males = 30%, females = 28%, unknown = 42%). However, when looking at the percentages of females and males self-awakeners within each single paper, several studies reported a higher percentage of females compared to males (n = 5; f/m %: 67/33; 60/40; 75/25; 55/45; 78/22), while only two papers reported the opposite pattern (n = 2; f/m %: 22/78; 22/78), and one paper only included male participants (n = 1; f/m %: 0/100).*”

36. Table 2 legend: rather long. Instead I propose to explain some of the terms in the column headers or in the cells (e.g. instead of using the abbreviation “E” just write “events”)

We abbreviated the table caption and indicated some of the abbreviations directly in the columns, as suggested.

37. If using the MEQ and not the Munich Chronotype Questionnaire, I would rather refer to circadian preference and not chronotype. These two are slightly different concepts.

In accordance with reviewer’s suggestion we refer to MEQ results as circadian preference and not chronotype throughout the text.

38. Fig. 3: I would suggest to increase the amount of ticks of the y-Axis, e.g. 0,2,4,6,8 etc to facilitate reading the values

Figure 3 has been updated as suggested, please see the detailed response above.

Discussion:

39. Line 349: I would suggest to report the actual number of participants, not just more than 700

We now report the exact number of participants (715) at line 386: “*We retrieved 17 papers, written in English, testing a total of 715 participants*”.

40. Line 351-352: “This result suggests self-awakening as a relatively understudied […] phenomenon”. Which result suggest this? It reads like the 17% but this does not make sense. Please clarify.

The sentence has been rephrased as follows (lines 388-389): “*The paucity of peer-reviewed papers, as well as the low calculated percentage of self-awakeners, are indicative of self-awakening as a relatively understudied and moderately rare phenomenon.*”

**Reviewer #2:** This review summarized 17 studies examining self-awakening and proposes a screening questionnaire for self-awakeners. Out of 715 total participants, 125 (17%) reported being self-awakeners, with an average age of 23.24 years and a slight predominance of males compared to females. 9 out of the 153 Dutch students are possible self-awakeners. These results reveal that studies and findings on self-awakening are a relatively rare phenomenon.

Although the topic is intriguing, several serious concerns arise and are listed below:

We thank the reviewer for the constructive and careful reading of our manuscript. Please find the in-line responses below.

1.I would delete the part on lucid dreaming lines 62-67.

We thank the reviewer for this suggestion. However, we believe that the phenomenon represents a good example of regained volition during periods, in which volition is not supposed to play a role. Moreover, reviewer 1 asked to elaborate more on this issue. For this reason we decided to maintain this part in the text.

2.Line 76 can’t it be a learned behavior and/or circadian rhythm issue?

We absolutely agree with the reviewer. In the case of habitual self-awakening the interaction between circadian rhythm and learned behaviour may explain the phenomenon. We specified at lines 80-82: “Third, self-wakening itself needs to be disambiguated: self-awakening might be habitual (i.e., a person waking up every day at the same time), in which case the interaction between circadian rhythms and learned behaviour are the driving factor, or can be induced by volition (Hayashi et al., 2010). This latter phenomenon represents the topic of interest here.”

3.Lines 72-78 need references.

We added the reference to the review paper by Hayashi et al (2010) in accordance with the reviewer’s suggestion (line 81).

4.Line 108 given your proposed taxonomy which type of self-awakening is being studied? This should be specified.

We thank the reviewer for this suggestion that allows us to better specify our aim; we did so at lines 114-115: “The aim of this article is to review the scientific literature on self-awakening intended as an ability in line with the taxonomy reported in Fig. 1.”

5.Lines 126-128 search terms cover aspects beyond the self-awaking ability.

Because not all papers, especially the older ones, would explicitly refer to “self-awakening ability”, we used broad search terms to maximise our chances to capture all the relevant papers. All the results were carefully screened to verify that they indeed covered the self-awakening phenomenon. Non relevant papers were excluded. We refer the reviewer to the newly added PRISMA flowchart, detailing the selection steps.

Also, if different languages were allowed in the search, what were the search terms to prevent bias? Please note, Lines 214-215 only mention English papers and abstracts.

Thank you for allowing us to clarify this aspect. We did not explicitly search for other languages, as translating the key terms in all possible and reasonable languages would have been impossible. Rather, we did not explicitly restrict the database searches to publications written in English. As peer-reviewed manuscripts in language other than English often include an English version of the abstract or title, our search would still have captured these publications. However, we only retrieved publications written in English (as reiterated at lines 243-244). This means that either this is the only language in which publications on self-awakenings were produced OR that publications in other languages did not fulfil our database search criteria. Still, we do agree that this point may be debatable, and we therefore rephrased the sentence concerning publication bias as follows (lines 140-143): “*We allowed the search to include manuscripts written in languages other than English; however, only English studies were retained in the final set of suitable papers based on the criteria outlined in the following section.*”

6.Line 120-121 an update of the literature is needed.

We repeated the research on October 6th, 2022, as also requested by Reviewer 1. As mentioned in response to #R1 comment, one additional paper was retrieved (S. Malloggi, F. Conte, B. Albinni, G. Gronchi, G. Ficca & F. Giganti (2022) Sleep and psychological characteristics in habitual self-awakeners and forced awakeners, Chronobiology International, 39:4, 547-556, DOI: 10.1080/07420528.2021.2003375). The search date has been updated in the manuscript. However, please note that this paper targeted habitual self-awakeners; as stated by the authors “participants were classified as habitual SAs if they answered “I never use external devices” to the first question and/or “I always wake up before the alarm goes off” to the second. Based on our taxonomy (see Fig. 1 in the text), this is a different phenomenon compared to self-awakening ability, which is the focus of our manuscript. For this reason, the paper was not included in the systematic review. It was, however, cited in the manuscript when we explicitly mention habitual self-awakening (e.g., line 399).

7.Line 131 was not elaborated on in later sections

Thank you for spotting this inconsistency; we clarified the sentence as follows (lines 138-140): *“Peer-reviewed and non-peer reviewed materials (e.g., conference abstracts appearing in Special Issues) were considered to avoid publication bias; yet only full manuscripts were retrieved from the database search.”*

8.Line 143 Quality appraisal – the applicability, hence validity, of the tool is very questionable given that it is designed for cohort studies. How many of your study designs were cohort studies?

Thank you for pointing this out. The CASP instrument is routinely and widely employed to evaluate studies to be included in systematic reviews, as most questions are extremely generic and can be applied to several types of study (only a few items explicitly refer to the extension of the follow-up). As mentioned in the manuscript, the studies we selected employed extremely varied methodologies, and we needed a broad instrument, such as CASP, to be able to cover this range.

9.Line 159 Was there any forward/backward translation done?

The test was originally created in Italian by the first and last authors (LV and AG), and translated into English. The translated version was then checked by two native speakers of American English and by several colleagues who daily use English as their main working language. The final version was then re-translated in Italian by the other co-authors.

Foremost if this was applied at Maastricht university, was a Dutch version used?

No, we did not use a Dutch version, as all the students were enrolled in the English track. Most students in this track come from different countries (both European and non-European) and did not speak Dutch at all. This is specified at lines 183-185: “*The questionnaire was administered to 164 students at Maastricht University (Maastricht, the Netherlands); all students were enrolled in the English track and used English daily as their main study language.*”

Also, what are the psychometric properties of the items selected from the literature, and the SAQ as well? The tools from which the items were selected should be discussed in the Introduction or even Methods. The re-using of items from the literature needs to be based on their psychometric property and not merely face validity.

For the test battery, we only selected full tests and questionnaires, and never single items. Most of these tests are routinely employed in clinical and research settings in their entirety, and their psychometric properties are published. We have now added more information about the tests in the Supporting Information.

Regarding the SAQ, we would like to stress that we did not intend to provide a full validation, but merely suggest some items that could be considered for the development of a potential screening tool. As the reviewer also noted, our sample - albeit quite large - would not be appropriate for a full-blown validation. More importantly, a true validation of this questionnaire would also be complemented by experimental data involving *objective* sleep validation methods (such as actigraphy or polysomnography). This step is necessary to verify that participants can, indeed, wake up spontaneously at their desired time. Without these additional steps, SAQ remains merely a proposal and a first step to stimulate further research.

10.What is the purpose of an Italian version?

Most of the authors involved in the current study (6 out of 7) are Italian, and five out of seven currently work in Italy. We reasoned that an Italian version would have been necessary to conduct further studies on self-awakening. This is especially the case as most authors often work with elderly populations and other participants whose native language is Italian and who may not be familiar (enough) with English.

11.Line 171-177 was any randomization applied?

No, we did not randomise the order of presentation of the tests in the battery.

12.Line 183 If SAQ contains 5 questions, and each item is scored as a 0 to 3, how can the maximum score be 13? This is misleading, especially when reading Table 1. A justification for the scoring method is needed. A report of several psychometric properties for the SAQ is lacking.

We refer the reviewer to Table 1 in the Supporting Information (column: assigned score), for a description of the score assigned to each item.

Regarding the psychometric properties of SAQ, we would like to stress that we had no intention of providing a validation of the questionnaire, for which a much bigger sample and additional objective methods would be required (see sections about the limitations of the study, lines 506-510).

13.How does the response of Q3 “No, just in the morning before the alarm clock” discriminate sufficiently toward self-awaking ability versus habitual self-awakening? For 58% of the sample, this is the answer. Responses like Q3 prime different interpretations throughout your questionnaire, which will affect the validity and reliability of your SAQ.

We thank the reviewer for their comment. We would like to emphasise that a single item does not, in itself, discriminate between a self-awakener and a non-self-awakener. It is the combination of scores (please see response to #Rev1, above), which together contributes to a well-rounded description of a probable self-awakener. For this reason, we set the cut-off at 11 points; this cut-off derives from the sum of all items with the highest scores; we reasoned that a self-awakener would have to respond to all items in a certain way (except for question 4, which allows multiple responses based on the existing literature) to be categorised as such. We would also like to stress that the current version of SAQ is simply a preliminary proposition, which is bound to be further investigated and objectively validated before it can be effectively used as a screening questionnaire.

14.A power calculation of the needed sample size is required because this study is underpowered. The literature estimate is 17% whereas your sample has 6%.

This is a rather interesting and important point. Unfortunately, the literature review included studies which varied immensely with respect to their theoretical definition as well as their methodological approach to the study of self-awakening. Some studies are themselves undersampled, with only 10 participants, and report much higher percentages as compared to studies with a higher number of participants (see, for example, Ikeda & Hayashi, 2010 - 90% SA as compared to Hawkins & Shaw, 1990 - 10%). Because of this variety, a calculation of the needed sample size would be highly unreliable. Even more importantly, we did not have an objective validation for our questionnaire, nor was this our aim. We stress in the limitation section that this should be a necessary next step for future studies.

15.The MANOVA is not valid and should be discarded. Lines 308,311, 312 etc , no Bonferroni correction?

We removed the MANOVA analysis, and the corresponding discussion of this result, from the manuscript.

16.Information is needed on the validity/reliability of the test battery applied to Dutch students.

Since all the students involved in the study were part of the English track of the Maastricht University we used the English version of all the employed tests. Unfortunately also very specific literature using some of these tests in the Dutch population does not refer to normative data in the Dutch language (as an example for the ISI see Baka et al. Cost-effectiveness of Guided Internet-Delivered Cognitive Behavioral Therapy in Comparison with Care-as-Usual for Patients with Insomnia in General Practice. Behav Sleep Med. 2022). When available, the requested information has been reported in the Supporting Information, section “3. Description of battery tests and their scores”.

17.Is Line 367 not contradictory to your purpose, i.e., creating the SAQ? Namely, is a screening tool not an outcome measure?

The reviewer raises an important point. Indeed, SAQ is intended as a screening tool to pre-select participants who may be prone to self-awakenings. As shown by our review and our own preliminary results, self-awakeners may be relatively rare in the general population; thus, by randomly inviting people in a sleep laboratory, the probability of finding a self-awakener is rather slim (see also #rev1’s comments and our responses in this regard). With SAQ, our aim is to maximise the chances of selecting suitable participants; thanks to this pre-selection, researchers interested in self-awakening may be facilitated in finding suitable candidates for their studies, which, we insist, would have to adopt objective methods (such as PSG) to confirm the occurrence of self-awakenings. We reinforce this suggestion in the abstract (“*(...) while providing a useful tool and empirical suggestions for future experimental studies, which should ideally employ objective measurements*.”) as well as in our conclusion (*“Future studies employing objective measurement such as EEG, PSG, and actigraphy, with the help of a comprehensive and innovative study design and a clear definition of self-awakening, are needed for a rigorous scientific study of this fascinating phenomenon.”*).

The importance of objective evaluation is now also underlined in the new section about the limitations of the study: “*Third, our study lacked an objective sleep evaluation to confirm whether the self-awakeners identified by SAQ were indeed capable of spontaneously waking up at a desired time. Objective sleep evaluation by means of PSG is of paramount importance in the study of self-awakening, and we strongly advise future studies to include these measurements.*”

Minor

Line 368, and Line 400 sentences need some English correction

The paragraph containing line 368 has been rephrased as follows (now lines 403-407): “In addition to issues related to definition, the current scientific approach to self-awakening is undermined by several methodological problems. For example, objective sleep assessments (e.g., PSG or actigraphy) are rarely used in the literature; yet, because they can reliably certify each participant’s wake-up time, they should be the first choice in the study of self-awakenings. Instead, subjective questionnaires and sleep diaries should only be used for screening purposes.”

Line 400 was rephrased as follows (now lines 458-466: “Notably, we observed a weak negative correlation between the scores obtained for SAQ, ISI, and PSQI. This relation suggests that self-awakeners (i.e., individuals with a high SAQ score) do not show signs of either insomnia (as evidenced by the low ISI scores) or low sleep quality (as evidenced by the low PSQI scores). This result complies with the definition of self-awakening as an independent phenomenon, unrelated to concomitant sleep disorders. While poor sleep quality or insomnia may also cause spontaneous awakenings, the presence of these disorders should constitute an important exclusion criterion when studying self-awakening.”

Line 382-383, line 405-410 is speculative, and so is Figure 4.

We rephrased as follows to make more clear that these are our interpretations/suggestions:

- Line 433-434: “*However,* ***we speculate****, the rather low percentage observed when applying the proposed criteria* ***may be indicative*** *of a naive conception of the phenomenon that does not adhere to the proposed definition.*”
- Lines 471-476: “*The investigation of self-awakening is rather relevant, because it might represent an interesting model for studying specific cognitive processes even in states of minimal or absent consciousness.* ***We propose that*** *three basic cognitive operations* ***may be*** *involved: (i) the encoding and storing of the target time, which must be readily accessible; (ii) the comparison between actual time and target time, which presumes the existence of a psychobiological ultradian timekeeper; (iii) the restoration of volitional control during sleep to wake up (****Fig. 4****)*.”
- Figure 4, caption: “*Fig. 4.* ***Proposed s****chematic model of the cognitive operations* ***potentially*** *involved in self-awakening.*”
