# Supplementary material for: Sleeping with time in mind? A literature review and a proposal for a screening questionnaire on self-awakening
Source: PLoS One. 2023 Mar 23;18(3):e0283221. doi: 10.1371/journal.pone.0283221 (PMC10035927; doi:10.1371/journal.pone.0283221)
Supplement: Attachment — Submitted filename: R2_response_to_reviewers.docx [file pone.0283221.s003.docx]

Dear Dr. Verga,

Thank you for submitting your manuscript to PLOS ONE. After careful consideration, we feel that it has merit but does not fully meet PLOS ONE’s publication criteria as it currently stands. Therefore, we invite you to submit a revised version of the manuscript that addresses the points raised during the review process.

==============================

Please include the points raised by Reviewer 1 in the paper – not just in the rebuttal.

==============================

We look forward to receiving your revised manuscript.

Kind regards,

Manuel Spitschan

Academic Editor

PLOS ONE

We sincerely thank the academic editor and the editorial team for handling our manuscript, and the anonymous reviewer for their careful evaluation of our work. We have revised the manuscript to include the points raised by Reviewer 1; please see the point-by-point responses below where we detail the changes introduced in this revision. Line numbers refer to the tracked-changes version.

Journal Requirements:

Please review your reference list to ensure that it is complete and correct. If you have cited papers that have been retracted, please include the rationale for doing so in the manuscript text, or remove these references and replace them with relevant current references. Any changes to the reference list should be mentioned in the rebuttal letter that accompanies your revised manuscript. If you need to cite a retracted article, indicate the article’s retracted status in the References list and also include a citation and full reference for the retraction notice.

We are not aware of any retraction concerning the papers in our Reference list. No items were newly added or removed from the reference list.

Reviewer's Responses to Questions

**Comments to the Author**

6. Review Comments to the Author

Reviewer #1: Thank you very much for the careful revision of your article and integrating my suggestions. I recommend publication based on the condition that you simply add some of your answer to my questions also to the document since I believe that these might also help other readers to better understand your article. These include the following:

We thank the reviewer for the positive and careful evaluation of our work. We have revised the manuscript to include the suggested changes. Please see below for the point-by-point responses.

- the explanation about power analysis and why recruitment stopped (point 9) should also be included in the methods

Thank you for this suggestions, we added the following explanations:

- About the recruitment method, at line 196-200: “Interested participants could either contact the experimenters directly via email or could select the study from those available on the SONA system, a participant pool management system used by many universities (<https://www.sona-systems.com/>). In SONA, interested students can easily find and enroll in ongoing studies in exchange for university credits or cash. All the participants in the current study were compensated with university credits.”
- About the power analysis, at lines 189-193: “Given the paucity of previous work on self-awakening and the exploratory nature of our study, we did not perform an a-priori power analysis to estimate an appropriate sample size. Yet, a number of participants > 150 can be considered a reasonable sample size for most types of analyses, especially for simple statistics as the ones performed in the current study.”
- About why the recruitment stopped, at lines 199-200: “Recruitment stopped when no further participants would sign up for the experiment after 2 months since the enrolment of the last participant.”

- maybe also include the description of point 10

We added the requested information at lines 217-226: “We set the cut-off to define a self-awakener at 11/13, based on our theoretical definition of self-awakener as well as on the score distribution. More specifically, the combination of the answers and scores needed to identify a self-awakener was as follows:

·	Question 1: “yes”, 1 POINT

·	Question 2: “only on nights before important events”, 3 POINTS

·	Questions 3: “No, just in the morning before the alarm clock”, 3 POINTS

·	Question 4: stratified answer, 1, 2, or 3 POINTS

·	Question 5: “When you have to wake up at an unusual time”, 3 POINTS

Thus, the minimum score needed to be defined as a self-awakener is 11, with some variability granted by Question 4.”

- Fig. 2 is very nice, thank you for adopting my suggestions. I still miss the different sample sizes for both self-awakeners and non self-awakeners; even though they don’t change, as you suggest, one has to go back and consult somewhere in the text

We apologize for missing out on this request in the previous revision. We now added the sample size in the figure caption, line 345: “Fig. 3. Differences between Self-Awakeners (n = 9) and non-Self-Awakeners (n = 144) by test scores”.

- point 20: thanks for adding this information to the discussion, this improves it

Thank you for the positive feedback.

- point 28: I guess my point was to specify the study design (i.e. observational study) instead of defining it by what it is NOT, i.e. non-experimental

We apologize for the misunderstanding; we added the information at line 149: “they were excluded if they were i) non-experimental (e.g., observational) “

- point 30: please add this information about translation in the method section

We added the details about SAQ translation at line 175-179: “SAQ was originally created in Italian by the first and last authors (LV and AG) and subsequently translated into English. The English version was checked by two native speakers of American English and by several colleagues using English daily as their main working language. The final version was then re-translated in Italian by the other co-authors.”
